# Evaluation of Anogenital Distance and Anti-Müllerian Hormone Plasmatic Concentration as Potential Phenotypes to Predict Reproductive Performance in Holstein Heifers

**DOI:** 10.3390/vetsci11100495

**Published:** 2024-10-12

**Authors:** Lucía Vidal, Jacobo Álvarez, Uxía Yáñez, Juan Caínzos, Rodrigo Muíño, Juan J. Becerra, Ana I. Peña, Luis A. Quintela, Pedro G. Herradón

**Affiliations:** 1 Unit of Reproduction and Obstetrics, Department of Animal Pathology, Faculty of Veterinary Medicine, Universidade de Santiago de Compostela (USC), 27002 Lugo, Spain; lucia.vidal.diaz@rai.usc.es (L.V.); jacobo.alvarez.torres@rai.usc.es (J.Á.); rodrigo.muino.otero@usc.es (R.M.); juanjose.becerra@usc.es (J.J.B.); ana.pena@usc.es (A.I.P.); luisangel.quintela@usc.es (L.A.Q.); garcia.herradon@usc.es (P.G.H.); 2 ABS Progenex, Calle Calidad 34, 28906 Getafe, Spain; juan.cainzos@genusplc.com; 3 Instituto de Biodiversidade Agraria e Desenvolvemento Rural (IBADER), Universidade de Santiago de Compostela (USC), Lugo University Campus s/n, 27002 Lugo, Spain

**Keywords:** anogenital distance, Anti-Müller hormone, fertility, reproductive phenotype, heifer

## Abstract

**Simple Summary:**

The profitability of dairy farms primarily depends on the productive and reproductive performance of cows. However, the progressive decrease in reproductive performance in dairy cattle has led to the search for new potential phenotypes to keep the most profitable animals. Consequently, this study aimed to assess whether anogenital distance or anti-Müllerian hormone plasmatic concentrations could be used as predictors of future reproductive performance in dairy cattle. The anogenital distance was measured in 566 heifers from 9 dairy farms in Galicia (Spain), and blood samples were collected. Our results showed that animals with short anogenital distance were inseminated, became pregnant, and calved earlier than animals with long anogenital distance. Additionally, it was verified that this measure showed little variation with age. However, no association was found between anti-Müllerian hormone plasmatic concentration and anogenital distance or reproductive performance, underlining the need for further studies with larger sample sizes.

**Abstract:**

Anogenital distance (AGD) is a marker of the degree of prenatal exposure to androgens in multiple species, and it has been suggested that there is an inverse association between AGD and fertility. Therefore, the aim of this study was to determine the usefulness of AGD and anti-Müllerian hormone (AMH) concentrations, an indirect marker of the follicular population, as predictors of future reproductive potential in Holstein cattle. The AGD was measured in 566 females from 9 dairy farms in Galicia (Spain). A group of 172 females underwent a second measurement 9 months after. Additionally, data on the age at first insemination (1stAI age), number of AI (AI-PREG), age at first pregnancy (1stPREG age), age at first calving (1stCAL age), and calving–pregnancy (CAL-PREG) and calving–calving (CAL-CAL) intervals were collected. Blood samples were collected from 80 heifers to determine AMH concentrations. Our results showed that AGD varied minimally with age, and that cows with short AGD had earlier 1stAI age, 1stPREG age, and 1stCAL age (*p* < 0.05) than cows with long AGD. No significant differences were observed for the CAL-PREG and CAL-CAL intervals. Additionally, no significant association was found between AMH concentration and AGD or reproductive parameters. Consequently, the results suggest the possibility of using AGD as a marker of future reproductive performance in Holstein heifers. However, there was insufficient evidence to associate AMH concentrations and reproductive performance, underlining the need for further studies with larger sample sizes.

## 1. Introduction

The reproductive success of animals is one of the main determinants of the profitability of dairy farms. In this way, each day that a cow remains non-pregnant after the voluntary waiting period reduces the amount of milk produced and its productive milk yield [1]. The reproductive performance of dairy cows has progressively decreased during the second half of the 20th century, reaching its minimum in the late 1990s [2,3]. Consequently, fertility problems have become common in high-producing herds, reducing the productive lifespan of animals.

The annual replacement rate varies significantly among farms, mainly depending on the productive aptitude of the cows. In dairy farms, this value may reach 25–35% [4]. In order to maintain the size of the farm, it is necessary to maintain an adequate number of heifers. These should be selected according to their productive and reproductive potential and managed appropriately to ensure that their first calving occurs at around 24 months, aiming to reduce production costs and shortening the generation interval [5].

Regarding the conventional parameters used to measure fertility (calving intervals, calving–gestation interval, percentage of pregnant cows and number of inseminations required to achieve gestation), it is known that their heritability is less than 5% [6], and that they depend on many other circumstances other than genetics, such as feeding, management, and environmental conditions, among others. For this reason, efforts are made to select the phenotypic characteristics related to fertility that are easy to determine, that are not subject to environmental modifications, and, if possible, that have high heritability. The ideal phenotypes would be those that can be easily determined during the early postnatal life stages and that allow predicting the future reproductive behavior of the female during her adult life [7].

One of the possible phenotypic characteristics that could be used as selection criteria for heifers is the anogenital distance (AGD) [8,9]. This parameter has been defined as the distance separating the anus and external genitalia in males and females. It initially emerged as an anthropometric value used to recognize the effect of endocrine disruptors on the development of human reproductive organs [10]. According to Callegari et al. [11], in the human species, AGD refers to the separation between the center of the anus and the base of the clitoris (AGDc) or the dorsal commissure of the vulva (AGDv). Different studies have shown the existence of a correlation between elevated AGD values with lower female fertility [12,13]. However, in the human species, it has been proposed that the ratio between the two measures has a higher correlation with fertility [14] and reduces the variations between breeds observed when using the two previous measures [15].

In the cattle, anogenital distance is defined as the measure of the separation between the center of the anus and the base of the clitoris (AGDc) [16]. This phenotype has a normal distribution and relatively high variability, repeatability, and heritability, estimated to be 0.37 by Gobikrushanth et al. [17] or between 0.23 and 0.29, depending on the age at which the measurement is made [18]. In addition, it shows an inverse correlation with fertility, is easy to define, and can be obtained simply and inexpensively at any time from the birth of a female. This makes it an ideal candidate to be used for genetic selection. Recently, following the same criteria already used in the human species, two other parameters related to anogenital distance have been defined: the distance between the anus and dorsal commissure of the vulva (AGDv) and the AGDc/AGDv ratio [19].

In this way, a study conducted with Canadian Holsteins has negatively correlated this distance with fertility during the first and second calving [8]. The authors observed that, for every 1 mm increase in AGDc with respect to the median, the probability of gestation at first insemination decreases by 1.9%. Thus, those females with a shorter distance recovered postpartum ovarian activity earlier; had estrus of greater intensity and duration, being more likely to be accompanied by ovulation; and had higher progesterone concentrations at 7 days post-insemination. In addition, the percentage of pregnant cows at 150 and 250 days of lactation was lower in cows with greater anogenital distance. On the other hand, results from a follow-up study by Gobikrushanth et al. [17] indicated that there was no association between AGD and fertility in over 1180 Irish Holstein-Friesian cows, as there was no variation in the number of inseminations or conception rate between cows with short or long AGD. These results indicate that the association between AGD and reproductive performance may be affected by other traits such as genetic makeup or type of production system.

Another possible structural indicator of a cow’s fertility is the antral follicle count (AFC), a parameter that has moderate heritability (0.31) and high repeatability (0.85 to 0.95) in dairy cows [20,21,22]. The primordial follicles are considered as the ovarian reserve, such that each female is born with a certain number of primordial follicles that will develop throughout her life. In terms of quantitative results, such as the aspiration of oocytes intended for in vitro production, cows with higher AFC have advantages [23]. On the contrary, it has been reported that cows with lower ovarian reserves have a shorter productive life [20], lower gestation rates, longer calving–gestation intervals, and more services per conception [22]. Nevertheless, it is worth noting that several studies, mainly involving *Bos indicus* cattle, have described that females with low AFC present better results during artificial insemination programs [24,25]. However, it is difficult to determine the AFC, since both adequate equipment and trained personnel are necessary.

Due to this problem, multiple studies have indirectly determined the population of 3 to 7 mm antral follicles [26] through serum levels of anti-Müller hormone (AMH), the latter also being considered as a marker of ovarian response to superovulatory treatments [27], of a female’s fertility [28] and the length of her productive life [20]. The AMH is a dimeric glycoprotein synthesized by Sertoli cells of the fetal testis, which is involved in sexual differentiation during development. This hormone causes the regression of the paramesonephric duct in the male fetus, the formation of the mesonephric duct, and the differentiation of the male reproductive system [29]. In contrast, during female fetal development, there is no production of AMH. Its expression in females has been studied in numerous species such as rodents, ruminants, equids, companion animals, and even humans. AMH is produced in all of them only by the granulosa cells of the antral follicles, the porcine species being the only one that also expresses this hormone in the theca cells of the preovulatory follicles and in the corpus luteum [29].

Like AFC, AMH is maintained in stable circulating concentrations throughout the reproductive life of the cow [30]. This fact allows measurement of circulating concentration at any stage of the estrous cycle or postpartum period to test associations with fertility outcomes [8]. Despite the high repeatability of plasma AMH concentrations in individual animals, there is a high level of variation in concentrations between cows, with numerous factors affecting such variation, such as breed, nutrition, age, or number of lactations [30].

Additionally, the AMH is also associated with measures of cow fertility. Ribero et al. [28] observed that cows with low AMH concentration had a lower pregnancy rate after estrus and, among pregnant cows, a higher number of abortions between 30 and 65 days of gestation. Another advantage of using AMH to predict female’s fertility is that its genomic heritability has been estimated to be between 0.36 and 0.43, much higher than that of other reproductive traits [31], which range between 0.01 and 0.13 [30]. Therefore, although AMH is influenced by other factors, given the correlation seen between AMH and fertility, the hormone might be a more reliable selection criterion than using other reproductive traits.

Finally, in the study conducted by Jiménez-Krassel et al. [20], a positive association was found between AMH concentration in Holstein heifers and their productive life. These females were monitored during gestation, calving, and subsequent lactations, observing that those with lower concentrations completed a lower number of lactations, with shorter productions compared to those with higher concentrations. These results point to AMH as a possible predictor of future profitability of the females, allowing early determination of their viability in the farms.

Considering this, the objective of the present study was to determine the use of AGD and AMH plasma concentration as predictors of the future reproductive potential of Holstein heifers.

## 2. Materials and Methods

### 2.1. Animals

This study was conducted on 9 dairy farms located in Galicia (Northwest Spain). Farms were selected according to their willingness to participate and having their productive and reproductive data collected using on-farm software (Gando Nuevas Tecnologías, Ferrol, Spain). The mean number of animals per farm was 443 (96–896). Regarding their age, 64% were milking cows, and 36% were heifers. The mean culling and replacement rates were 29.70 and 32.30%, respectively.

Animals were housed in free-stall facilities under intensive management. They were fed a total mixed ration based on grass and corn silage, supplemented according to the energy needs, and provided ad libitum access to water. All farms had a conventional milking parlor, and cows were milked twice a day. The mean standardized production was 11,153.67 L, with a mean of 200.17 days in milk (DIM) and a somatic cell count of 343.621 cell/mL. Reproductive evaluations were performed periodically by an experienced veterinarian, depending on the farm size (from twice a month to once a week), to assess for postpartum health and pregnancy diagnosis, using transrectal ultrasonography. Artificial insemination was performed following heat detection, either visually or with the aid of monitoring devices.

### 2.2. Anogenital Distance Measurement and Reproductive Parameters

**Figure 1:**
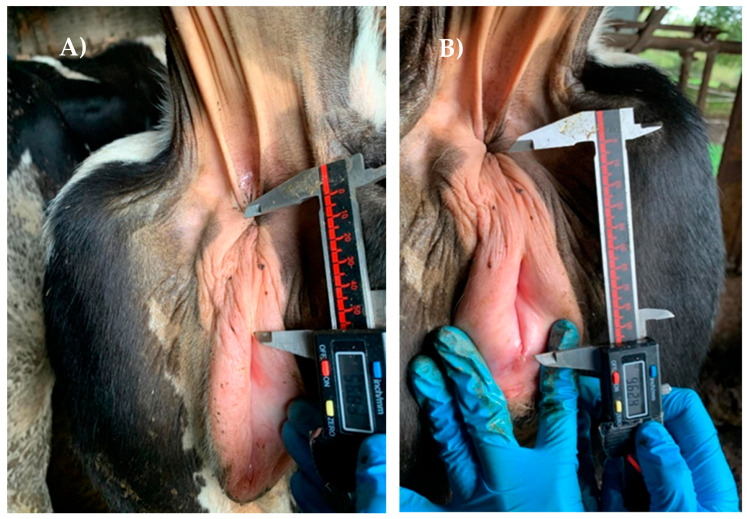
Measurement of the anogenital distance between the center of the anus and (**A**) the dorsal corner of the vulva or (**B**) base of the clitoris, using a digital caliper.

The study was conducted in accordance with the European and Spanish Regulations for the protection of animals used for scientific purposes (Directive 2010/63/EU, RD 53/2013). The AGD was measured using a digital caliper (Bricodi 0–150 mm) following the protocol described by Gobikrushanth et al. [16]. The AGDv was measured from the center of the anus to the dorsal corner of the vulva (Figure 1A). On the other hand, the AGDc was measured from the center of the anus to the base of the clitoris (Figure 1B). Additionally, the anogenital index was calculated as AGDv/AGDc*100.

Measures were taken with the animals tied. Females with abnormalities in the perineal region, such as inflammation or lacerations, were excluded. All measurements were collected by the same person, with the aid of an assistant that held the tail aside for ease of access to the anogenital region and limit the movement of the animal. Data for AGDv and AGDc were recorded for 121 heifers. After a preliminary analysis (see Pearson’s correlation in the Results section), the AGDc was measured in a total of 566 females. Additionally, in a group of 172 animals, a second measurement was performed 9 months after the first one. In all cases, the age of the animals was collected.

Data for productive and reproductive parameters were obtained from the on-farm software Gando. The reproductive parameters considered were age at first insemination (1stAI age), number of AIs needed to become pregnant for the first time (AI-PREG1), age at the beginning of the first pregnancy (1stPREG age), age at first calving (1stCAL age), number of AI needed to become pregnant for the second time (AI-PREG2), calving to conception interval (CAL-PREG), and calving to calving interval (CAL-CAL). The CAL-CAL was calculated using the estimated calving date provided by the software.

### 2.3. Anti-Mullerian Hormone Determination

After measuring the ADG, blood samples (*n* = 80) were collected from the coccygeal vein using vacuum tubes with the anticoagulant EDTA (BD Vacutainer^®^, Becton, Dickinson and company, Franklin Lakes, NJ, USA). Samples were kept in refrigeration (<8 °C) for their transportation to the laboratory at the Faculty of Veterinary Science of the University of Santiago de Compostela (Lugo, Spain), and plasma was obtained within 2 h after collection by centrifugation at 1500× *g* for 20 min. Plasma was stored at −20 °C until analysis.

The plasmatic concentration of AMH was determined using a commercial ELISA kit (AMH Bovine ELISA, AnshLabs, Webster, TX, USA) following the manufacturer’s instructions. The assay was calibrated using 5 different recombinant bovine AMH vials, with concentrations ranging from 13 to 2200 pg/mL, with 11 pg/mL being the lower detection limit. Three control samples were checked in 24 replicas, with an intra-assay variation coefficient of 2.54%. Optical densities were measured using a microplate reader (Multiskan EX, Thermo Fisher Scientific Inc., Waltham, MA, USA).

### 2.4. Statistical Analysis

All analyses were conducted using IBM SPSS Statistics version 29.0.1.0 (IBM Corp., Armonk, NY, USA). Differences were considered significant at *p* ≤ 0.05, and tendencies were considered when *p* ≤ 0.10.

First, descriptive statistics and Pearson’s correlation analysis were performed to establish a preliminary relationship between the AGDc, AGDv, and anogenital index and the reproductive parameters. After assessing that the AGDc presented a more statistically significant correlation compared to the AGDv and anogenital index, animals were separated into three different groups depending on their AGDc: Short AGDc (58.65–85 mm), Medium AGDc (85.01–115 mm), and Long AGDc (115.01–140.48 mm). Afterwards, an ANOVA test was carried out, including the AGDc and farm as factors and the different reproductive parameters as dependent variables.

Finally, Pearson’s correlation analysis was carried out between the AMH concentrations and the different reproductive parameters, using the bivariant correlation procedure. To determine the effect of the AGDc on the AMH levels, an ANOVA test was performed, including the AGDc and farm as factors and the AMH levels as a dependent variable. Additionally, a dispersion diagram was displayed to establish the distribution of the AMH levels within the collective.

## 3. Results

The overall results for descriptive statistics for the characteristics of the farms and their reproductive parameters are displayed in Tables S1 and S2. Regarding the different measures for AGD, the mean values observed for the AGDc, AGDv, and anogenital index were 117.40 ± 14.56 mm, 59.68 ± 11.41 mm, and 51.25 ± 9.90 mm, respectively.

### 3.1. Correlation between the AGD and Reproductive Parameters

The results of Pearson’s correlation analysis (Table 1) showed a statistically significant positive correlation between AGDv, AGDc, and the anogenital index. However, considering reproductive parameters, no statistically significant correlations were observed for AGDv and AI-PREG1, 1stAI age, 1stPREG age, and 1stCAL age. On the other hand, the AGDc showed significant correlations with 1stAI age and 1stPREG age. As for the anogenital index, only a statistically significant correlation was observed for 1stAI age. Based on these preliminary results, we decided to continue the experiment using only the AGDc.

### 3.2. AGDc Distribution and Variation with Age

Descriptive statistics for the AGDc measures taken 9 months apart for the same animals are depicted in Table 2. Although the values collected during the second measurement were higher (117.40 vs. 99.43 mm), the average individual increase between both measurements was 0.051 mm (Table 2).

### 3.3. Relationship between AGDc and Reproductive Parameters

Regarding heifers, the mean AGDc was 76.21 ± 6.46 for the Short AGDc group, 101.14 ± 7.09 for the Medium AGDc group, and 120.23 ± 6.51 for the Long AGDc group.

Additionally, statistically significant differences were observed for 1stAI age between animals in the Short AGDc and Medium or Long AGDc groups (Table 3). Moreover, statistically significant differences were observed for 1stPREG age and 1stCAL age between cows in the Short AGDc and Long AGDc groups (Table 3). Similarly, numerical differences, although not statistically significant, were also obtained for AI-PREG1, as animals in the Short AGDc group needed less AI to become pregnant (Table 3). Finally, for those cows that became pregnant during their first lactation, numerical differences, although not statistically significant, were observed for CAL-PREG and CAL-CAL between animals in Short AGDc and Long AGDc groups (Table 4). In addition, cows in the Long AGDc group needed less AI to become pregnant a second time (Table 4). It should be noted that a statistically significant farm effect was observed for all reproductive parameters, except for AI-PREG2.

### 3.4. Relationship between AGDc and AMH Plasmatic Concentrations

**Figure 2:**
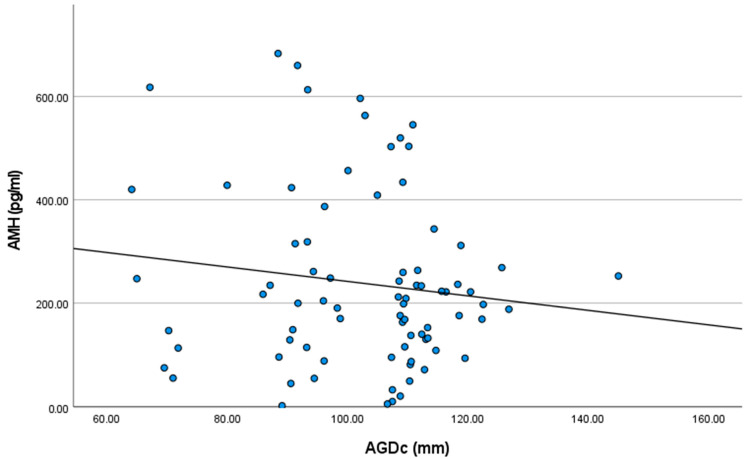
Results of Pearson’s correlation analysis between anti-Mullerian hormone plasmatic concentrations (AMH) and the anogenital distance (AGDc) in 80 Holstein heifers (R^2^ = 0.016; *p* > 0.05).

The mean AMH plasmatic concentrations were 263.101 ± 204.29, 240.30 ± 179.49, and 213.43 ± 55.25 pg/mL for the groups Short AGDc, Medium AGDc, and Long AGDc, respectively. Although decreasing HAM plasmatic concentrations were observed as AGDc increased (r = −0.128; Figure 2), no statistically significant correlations were observed.

## 4. Discussion

Our results showed that the AGDc may be a better indicator for future reproductive performance in heifers than AGDv or the anogenital index.

Regarding AGDc, the mean value observed in the current study was slightly lower than that observed by Beci et al. [19] in Holstein heifers, and considerably lower than those described by Gobikrushanth et al. [16,17] and Akbarinejad et al. [32] in Holstein cows. Considering this, it could be possible that the AGDc would be a repeatable measurement in animals with similar characteristics, as previously suggested [33], but could substantially differ with age. However, an increase of just 0.051 mm was observed in the current study, suggesting that the determination of the AGDc at an early stage, always before the first AI, could be of use in the selection of heifers with better reproductive potential, as it is minimally influenced by post-natal factors. These results do not agree with those reported by Beci et al. [19], who described considerable variation for AGDc, increasing by 3.8 mm per month. On the contrary, Carrelli et al. [8] stated that there were no significant differences regarding AGDc between heifers and primiparous cows. Additionally, in a later study, these researchers observed [33] that the AGDc in cows in their first, second, or third lactation only differed by 2, 1, and 1 mm, respectively, compared to heifers.

### 4.1. AGDc as Predictive Phenotype for Reproductive Performance

According to our results, there is a negative correlation between AGDc and reproductive parameters. In this way, heifers with short AGDc had lower 1stAI age, 1stPREG age, and 1stCAL age compared to heifers with long AGDc.

Heifers with short AGDc became pregnant approximately one month earlier than those with long AGDc. They also required a lower AI-PREG1 (2.17 vs. 2.60). Despite this, it was not possible to prove that the difference in AI-PREG1 between these categories was statistically significant. Instead, it has been shown that AI-PREG1 was highly influenced by the management carried out among the different farms, being affected by factors such as the efficiency of heat detection, seminal quality or the body condition score of the heifers at the beginning of the insemination.

In relation to these results, similarities have been found with the study performed by Akbarinejad et al. [32], where they did not obtain a statistically significant difference (*p* > 0.05) in the number of inseminations between categories. However, other studies, such as those presented by Gobikrushanth et al. [16] and Carreli et al. [33], did find a statistically significant difference in determining that females with long AGDc needed a higher number of inseminations per gestation. These results may be due to the fact that females included in the short AGDc group resume estrous activity in the first 50 days in milk have larger preovulatory follicles and higher ovulation rates [34].

Regarding multiparous cows, we observed that those with long AGDc had CAL-PREG and CAL-CAL values of 104.31 and 383.24 days, respectively. This represents an increase, in both intervals, of approximately 3 days compared to females with medium AGDc. However, as with the number of inseminations mentioned above, the difference was not statistically significant. On the contrary, a very strong association was found between CAL-PREG (*p* = 0.025) and CAL-CAL (*p* = 0.029) with the management carried out among the different farms, probably due to factors that vary between farms such as nutrition, environment, heat detection, gestation control, or diseases [35].

In line with the above, similar results have been found in the study conducted by Grala et al. [36], where primiparous cows with short AGDc presented a shorter CAL-PREG. Likewise, other studies [16] have found associations in second and third calvings, such that the percentage of gestations at first insemination in second-calving females was approximately 20% higher in cows with short AGDc compared to those with long AGDc.

Regarding multiparous cows, the lack of association we found between conception rates and AGDc could be due to other factors affecting conception, such as negative energy balance, higher incidence of early postpartum diseases (endometritis and retained placenta), or higher milk production by older cows [37].

Likewise, the lower reproductive performance observed in females belonging to the long AGDc group could be attributed to the effects of androgen exposure during the prenatal period [32]. It is the absence of androgen exposure in females during the period of sexual differentiation that prevents cranial migration of the genital tubercle, making the AGD shorter than in males [38,39]. Some studies in rats have shown that intrauterine exposure to some endocrine disruptors cause an increase in anogenital distance at birth [40]. We are not aware of any studies in cattle evaluating the effect of endocrine disruptors on AGD.

Our results may indicate that androgen exposure not only has structural consequences but also affects different regulatory systems such as neuroendocrine and metabolic systems [41]. Exposure of a female to high levels of androgens during prenatal life alters the expression of different receptors for steroids, cytokines, and apoptosis regulatory factors in the ovary [42,43]. In addition, prenatal androgen exposure could alter the regulatory effects of estradiol at the hypothalamic level by modifying tonic and cyclic luteinizing hormone (LH) discharges and attenuate the negative effects of LH secretion during the luteal phase [44]. Taking all these effects together, androgen exposure can lead to persistent follicles [45], anovulation, alterations of the estrous cycle [46], and reduced fertility [47].

Finally, when analyzing the effects of AGDc on the onset of second gestation (CAL-PREG and CAL-CAL), we could not observe statistically significant differences, which have previously been observed by other authors [33]. These differences are probably due to the limited number of data available, as a longer period is needed. It would also be interesting to know the possible correlation between AGDc and the milk yield, although this would require waiting until the animals finish their lactation. In this sense, a positive association (r = 0.041; *p* < 0.01) has been demonstrated between AGD and normalized production at 305 days [33]. This indicates that a selection of females with short AGD would result in lower yields; however, it is estimated that this loss in milk quantity is less significant compared to the gain in fertility that would result from the selection of animals by AGD [33].

### 4.2. Relationship between AGDc and Plasmatic AMH Concentrations

Our results did not show a statistically significant association between AMH plasma concentrations and the reproductive parameters. Additionally, to try to understand the mechanisms linking androgen exposure during prenatal life and reproductive performance, we have tried to detect the existence of an association between AGD and AMH levels. However, no statistically significant correlation was observed.

These results are in agreement with previous studies, which failed to demonstrate an association between AGD and AMH levels [48] or with AFC present in the ovary [36,48]. However, our results clearly differ from those obtained by Akbarinejad et al. [32], who observed that AMH levels were higher in cows with long AGDc, which could imply that the situation of cows is different from that of heifers. However, these same authors observed that females with higher AGD had lower reproductive performance.

In order to understand the mechanisms that relate androgen exposure during prenatal life and reproductive performance, it is necessary to relate this parameter to some functional characteristic that can be easily estimated from a phenotypic point of view. In this way, the observed correlation between AMH concentrations and AFC has allowed the development of a simpler and more objective procedure for donor selection or for predicting the fertility of a female [28] and the length of her productive life [20]. It is now known that AMH plays an important role in two stages of folliculogenesis. On the one hand, it inhibits the recruitment of primordial to primary follicles, such that the AFC is limited, thus avoiding premature depletion of the follicular reserve [29]. On the other hand, it inhibits the stimulatory effect of follicle stimulating hormone (FSH) on the growth of preantral and antral follicles, resulting in fewer follicles selected to grow to the preovulatory phase. The ultimate goal of these two functions lies in provoking optimal follicular development, limiting the final number of growing follicles, the AFC that will continue to grow, and regulating the follicles that will reach the preovulatory phase [29].

Considering this, it should be expected that the higher the AMH plasma levels, the higher the reproductive performance. Nevertheless, there is controversy about the association between these two parameters. Both a positive association [20,28] and no association at all [49,50] have been described, while no research reporting a negative association has been published. Although not significant, our results showed that heifers with short AGDc had higher AMH plasmatic concentrations compared to heifers with long AGDc (263.1 vs. 213.43 pg/mL), indicating a negative correlation between AGDc and AMH plasmatic levels. Taking into account that heifers with short AGDc had lower 1stAI age, 1stPREG age, and 1stCALage than heifers with long AGDc, further research is required to clarify whether there is actually an association between AMH concentrations and ADGc and reproductive performance, paying special attention to the sample size, which may have limited the statistical power in our study.

## 5. Conclusions

It can be concluded that the AGDc is negatively correlated with reproductive performance and could be useful for the prediction of the reproductive potential of Holstein heifers. Moreover, this measure is easy to define, can be obtained simply and inexpensively at any time from the birth of a female, and does not vary substantially with age. As for AMH concentrations, further research with a larger sample size is needed to clarify its association with both the AGDc and reproductive performance.

## Figures and Tables

**Table 1:** Results of Pearson’s correlation analysis including the anogenital distance determined from the center of the anus to the dorsal corner of the vulva (AGDv), from the center of the anus to the base of the clitoris (AGDc), the anogenital index (Index), the number of artificial inseminations needed to become pregnant (AI-PREG1), the age at first artificial insemination (1stAI age), the age at the beginning of the first pregnancy (1stPREG age), and the age at first calving (1stCAL age).

	AGDv	AGDc	Index	AI-PREG1	1stAI Age	1stPREG Age	1stCAL Age
AGDv	1	0.518 **	0.800 **	0.168	−0.162	0.019	0.040
AGDc	-	1	−0.090	0.070	0.139 **	0.140 **	0.092
Index	-	-	1	0.147	−0.227 *	−0.050	−0.015
AI-PREG1	-	-	-	1	0.039	0.059	−0.037
1stAI age	-	-	-	-	1	1.000 ***	0.008
1stPREG age	-	-	-	-	-	1	−0.009
1stCAL age	-	-	-	-	-	-	1

* *p* ≤ 0.05; ** *p* ≤ 0.01; *** *p* ≤ 0.001.

**Table 2:** Descriptive statistics (mean ± standard deviation, minimum and maximum) for the two measurements collected for the anogenital distance (ADGc1, AGDc2) and the increase observed after nine months.

	N	Mean ± SD	Minimum	Maximum
AGDc1 (mm)	172	99.43 ± 15.76	64.96	113.33
AGDc2 (mm)	172	117.4 ± 14.55	87.85	151.82
Increase (mm)	172	0.05 ± 0.03	0.00	0.12

**Table 3:** Descriptive statistics (mean ± standard deviation) and results for ANOVA test for age at first artificial insemination, (1stAI age), age at the beginning of the first pregnancy (1stPREG age), age at first calving (1stCAL age), and number of artificial inseminations needed to become pregnant for the first time (AI-PREG1) including data from 449 Holstein cows classified according to their anogenital distance (AGDc) into three groups: Short (58.65–85 mm), Medium (85.01–115 mm), and Long AGDc (115.01–140.48 mm).

	AGDc	N	Mean ± SD
1stAI age (months)	Short	55	13.37 ± 1.13 ^a^
	Medium	312	13.83 ± 1.66 ^b^
	Long	82	14.13 ± 1.49 ^b^
	Total	449	13.83 ± 1.58
1stPREG age (months)	Short	55	14.38 ± 1.95 ^a^
	Medium	312	15.36 ± 2.92 ^b^
	Long	82	15.60 ± 2.10 ^b^
	Total	449	15.29 ± 2.70
1stCAL age (months)	Short	55	23.64 ± 1.92 ^a^
	Medium	312	24.47 ± 2.84 ^ab^
	Long	82	24.75 ± 2.08 ^b^
	Total	449	24.42 ± 2.63
AI-PREG1 (n)	Short	55	2.02 ± 1.19 ^ab^
	Medium	312	2.11 ± 1.47 ^a^
	Long	82	2.48 ± 1.44 ^b^
	Total	449	2.17 ± 1.44

^ab^: Different letters within the same column indicate statistically significant differences.

**Table 4:** Descriptive statistics (mean ± standard deviation) and results for ANOVA test for number of AI needed to become pregnant for the second time (AI-PREG2), calving to conception interval (CAL-PREG), and calving to calving interval (CAL-CAL) including data from 111 Holstein cows classified according to their anogenital distance (AGDc) in two groups: Medium (85.01–115 mm), and Long AGDc (115.01–140.48 mm). No statistically significant differences were observed among groups.

	AGDc	N	Mean ± SD
AI-PREG2 (n)	Medium	82	1.83 ± 1.09
	Long	29	1.45 ± 0.69
	Total	111	1.73 ± 1.01
CAL-PREG (days)	Medium	82	100.41 ± 33.78
	Long	29	104.31 ± 36.03
	Total	111	101.43 ± 34.26
CAL-CAL (days)	Medium	82	380.33 ± 33.76
	Long	29	383.24 ± 36.64
	Total	111	381.09 ± 34.39

## Data Availability

Data can be provided by the corresponding author under reasonable request.

## Supplementary Materials

The following supporting information can be downloaded at https://www.mdpi.com/article/10.3390/vetsci11100495/s1: Table S1: Descriptive data for the composition and productive parameters of the nine Holstein farms included in the study. It displays the total number of animals present in the farms (Animals), the percentage of cows and heifers, culling (CR) and replacement rates (RR), mean parity, annual production (Annual Prod), standardized 305-day production (Standardized Prod), somatic cell count (SCC), and days in milk (DIM); Table S2: Descriptive data for the reproductive parameters of the nine Holstein farms included in the study. It displays the percentage of pregnant animals present in the farm (Pregnant), the mean age at first calving (Calving Age), the interval between calving and first artificial insemination (CAL-AI), the interval between calving and pregnancy (CAL-PREG), the interval between calvings (CAL-CAL), first service conception rate (FSCR), conception rate (CR), service rate (SR), and pregnancy rate (PR).
