# Supplementary material for: Evaluation of Anogenital Distance and Anti-Müllerian Hormone Plasmatic Concentration as Potential Phenotypes to Predict Reproductive Performance in Holstein Heifers
Source: Vet Sci. 2024 Oct 12;11(10):495. doi: 10.3390/vetsci11100495 (PMC11512217; doi:10.3390/vetsci11100495)
Supplement: Supplementary file 1 [file vetsci-11-00495-s001.zip › vetsci-3218179-supplementary.pdf]

Table S1. Descriptive data for the composition and productive parameters of the nine Holstein farms included in the study. It displays the total number of animals present in the farms (Animals), the percentage of cows and heifers, culling (CR) and replacement rates (RR), mean parity, annual production (Annual Prod), standardized 305-day production (Standardized Prod), somatic cell count (SCC), and days in milk (DIM).

Farm	Animals (n)	Cows (%)	Heifers (%)	CR (%)	RR (%)	Parity (n)	Anual Prod (L)	Stardardized Prod (L)	SCC (cell/mL)	DIM (d)
**1**	489	57.7	42.3	31.6	29.9	2.39	13427	11471	346.97	219.27
**2**	584	62.5	27.5	30.2	30.2	2.44	13711	11870	385.83	179.11
**3**	319	74.0	26.0	38.9	29.3	2.19	13661	10873	300.94	168.92
**4**	507	60.4	39.6	38.8	39.7	2.1	14990	11917	372.01	190.16
**5**	96	66.7	33.3	8.3	26.5	2.23	13999	11420	186.38	339.5
**6**	559	54.0	46.0	35.6	28.7	2.28	12950	11054	385.15	203.45
**7**	896	65.8	34.2	25.3	32.9	2.31	13637	11763	352.93	195.22
**8**	208	61.1	38.9	23.8	36.6	2.20	10528	9256	382.39	145.56
**9**	332	75.0	25.0	35.0	37.1	2.13	12669	10759	379.99	160.36
**Mean**	**443**	**64.1**	**34.8**	**29.7**	**32.3**	**2.25**	**13285.78**	**11153.67**	**343.62**	**200.17**

Table S2. Descriptive data for the reproductive parameters of the nine Holstein farms included in the study. It displays the percentage of pregnant animals present in the farm (Pregnant), the mean age at first calving (Calving Age), the interval between calving and first artificial insemination (CAL-AI), the interval between calving and pregnancy (CAL-PREG), the interval between calvings (CAL-CAL), first service conception rate (FSCR), conception rate (CR), service rate (SR), and pregnancy rate (PR).

Farm	Pregnant (%)	Calving Age (months)	CAL-AI (d)	CAL-PREG (d)	CAL-CAL (d)	FSCR (%)	CR (%)	SR (%)	PR (%)
**1**	48.6	25.03	79.13	151.79	413.43	28.02	25.92	57.33	14.86
**2**	49.1	22.46	70.29	102.98	397.70	47.75	40.99	73.43	30.17
**3**	42.4	25.39	70.53	137.63	441.18	21.81	23.73	68.00	16.13
**4**	45.4	26.46	86.77	137.83	435.85	35.36	21.64	62.93	13.62
**5**	60.9	26.33	80.71	170.22	449.00	19.23	28.89	58.25	16.83
**6**	57.3	23.26	78.20	132.52	411.85	30.11	34.52	59.74	20.62
**7**	52.9	24.10	78.02	144.62	418.21	24.23	29.16	48.43	14.12
**8**	49.6	20.17	71.50	97.17	404.30	47.57	51.10	48.79	24.93
**9**	49.0	24.44	94.10	127.10	403.83	45.07	41.10	45.59	20.39
**Mean**	**50.6**	**24.18**	**78.81**	**133.54**	**419.48**	**33.24**	**33.01**	**58.05**	**19.07**
